# Высокоселективный ингибитор натрий-глюкозного котранспортера 2 типа эмпаглифлозин как средство защиты головного мозга в условиях хронической недостаточности мозгового кровообращения (клинико-экспериментальное исследование)

**DOI:** 10.14341/probl13336

**Published:** 2024-09-15

**Authors:** А. В. Симаненкова, О. С. Фукс, Н. В. Тимкина, Д. А. Суфиева, О. В. Кирик, Д. Э. Коржевский, Т. Д. Власов, Т. Л. Каронова

**Affiliations:** Национальный медицинский исследовательский центр им. В.А. Алмазова; Первый Санкт-Петербургский государственный медицинский университет им. акад. И.П. Павлова; Национальный медицинский исследовательский центр им. В.А. Алмазова; Национальный медицинский исследовательский центр им. В.А. Алмазова; Первый Санкт-Петербургский государственный медицинский университет им. акад. И.П. Павлова; Институт экспериментальной медицины; Институт экспериментальной медицины; Институт экспериментальной медицины; Первый Санкт-Петербургский государственный медицинский университет им. акад. И.П. Павлова; Национальный медицинский исследовательский центр им. В.А. Алмазова; Первый Санкт-Петербургский государственный медицинский университет им. акад. И.П. Павлова

**Keywords:** сахарный диабет, хроническая недостаточность мозгового кровообращения, нейропротекция, ингибиторы натрий-глюкозного котранспортера 2 типа, эмпаглифлозин

## Abstract

**ОБОСНОВАНИЕ:**

ОБОСНОВАНИЕ. Хроническая недостаточность мозгового кровообращения является одним из частых осложнений сахарного диабета 2 типа (СД2) и приводит к инвалидизации больных. Ингибиторы натрий-глюкозного котранспортера 2 типа (иНГЛТ-2) доказали преимущества в отношении сердечно-сосудистой системы, однако их влияние на центральную нервную систему (ЦНС) исследовано недостаточно.

**ЦЕЛЬ:**

ЦЕЛЬ. Изучить влияние терапии эмпаглифлозином на функциональные и лабораторные параметры повреждения ЦНС у больных СД2 и в условиях эксперимента исследовать механизмы нейротропного эффекта препарата.

**МАТЕРИАЛЫ И МЕТОДЫ:**

МАТЕРИАЛЫ И МЕТОДЫ. В клиническую часть исследования включены пациенты с СД2 на монотерапии метформином (n=39). Пациенты с целевым уровнем гликированного гемоглобина (HbA1c) составили группу «МЕТ» (n=19), у пациентов с нецелевым уровнем HbA1c к терапии добавлялся эмпаглифлозин на 6 мес. (группа «МЕТ+ЭМПА», n=20). Здоровые добровольцы составили группу контроля (n=16). Изучался когнитивный статус и концентрация нейронспецифической енолазы (НСЕ) и легких цепей нейрофиламента (ЛЦН). В экспериментальном исследовании у крыс моделировался СД, и в течение 8 недель проводилось лечение эмпаглифлозином. Оценивали активацию микроглии при помощи анти-Iba-1 антител и морфологические изменения нейронов при окраске по методу Ниссля.

**РЕЗУЛЬТАТЫ:**

РЕЗУЛЬТАТЫ. Как в группе «МЕТ+ЭМПА», так и в группе «МЕТ», наблюдался когнитивный дефицит, по данным Монреальской шкалы когнитивной оценки (МОСА) (24,0 (23,0; 27,0) и 25,0 (21,0; 27,0) баллов) и Краткой шкалы оценки психического статуса (MMSE) (23,75 (23,0; 27,0) и 25,0 (21,0; 27,0) баллов). Терапия эмпаглифлозином привела к нормализации когнитивного статуса через 6 мес. (26,5 (24,0; 27,0) балла по шкале МОСА и 27,5 (24,0; 28,0) балла по MMSE). Исходно у всех пациентов имелось достоверное повышение НСЕ (3,60 (2,66; 3,76) нг/мл в группе «МЕТ», 3,22 (2,94; 3,54) нг/мл в группе «МЕТ+ЭМПА», 2,72 (2,13; 2,72) нг/мл в группе «Контроль») и ЛЦН (4,50 (3,31; 5,56) нг/мл в группе «МЕТ», 5,25 (3,75; 6,25) нг/мл в группе «МЕТ+ЭМПА» при 3,50 (2,25; 3,50) нг/мл в группе «Контроль»). Терапия эмпаглифлозином привела к достоверному снижению ЛЦН уже через 3 мес. (3,80 (3,25; 3,87) нг/мл), значимо не повлияв на уровень НСЕ. В эксперименте СД характеризовался увеличенным количеством активированных микроглиоцитов и деструктурированных нейронов и снижением числа нейронов с нормальной структурой. Терапия эмпаглифлозином сопровождалась уменьшением числа иммунопозитивных микроглиоцитов в СА1 зоне гиппокампа и увеличением числа структурированных нейронов.

**ЗАКЛЮЧЕНИЕ:**

ЗАКЛЮЧЕНИЕ. СД2 характеризуется развитием функциональных и биохимических изменений в ЦНС даже при удовлетворительном контроле гликемии. Терапия эмпаглифлозином оказывает нейропротективный эффект, проявляющийся улучшением когнитивного статуса и снижением уровня ЛЦН. Эмпаглифлозин уменьшает повреждение нейронов и снижает патологическую активацию микроглии.

## Abstract (en)

**BACKGROUND:**

BACKGROUND: Chronic brain dyscirculation is one of the frequent type 2 diabetes mellitus (DM) complications and leads to patients’ disability. Sodium-glucose co-transporter type 2 inhibitors (SGLT-2i) have been proven to have advantages for cardiovascular system, but their effect on the central nervous system (CNS) has not been studied enough.

**AIM:**

AIM: To study empagliflozin effect on CNS damage functional and laboratory parameters in patients with type 2 DM and, under experimental conditions, to investigate the mechanisms of the drug neurotropic effect.

**MATERIALS AND METHODS:**

MATERIALS AND METHODS: The clinical part of the study included patients with type 2 DM on metformin monotherapy (n=39). Patients with a target glycated hemoglobin level formed the “MET” group (n=19), in patients with a non-target glycated hemoglobin level empagliflozin was co-administered for the following 6 months (the “MET+EMPA” group, n=20). Healthy volunteers comprised the control group (n=16). The cognitive status and neuron-specific enolase (NSE) and neurofilament light chains (NLC) concentration were studied. DM was modeled in rats, thereafter the rats were treated with empagliflozin for 8 weeks. Microglia activation was assessed using anti-Iba-1 antibodies and morphological changes in neurons when stained by the Nissl method.

**RESULTS:**

RESULTS: Both in the “MET+EMPA” and the “MET” groups cognitive deficits were observed, according to the Montreal Cognitive Assessment (MOCA) (24.0 (23.0; 27.0) and 25.0 (21.0; 27.0) points) and the Mini-Mental State Examination (MMSE) (23.75 (23.0; 27.0) and 25.0 (21.0; 27.0) points). Empagliflozin therapy led to the cognitive status normalization after 6 months (26.5 (24.0; 27.0) points according to the MOCA scale and 27.5 (24.0; 28.0) points according to the MMSE). Initially, all patients had a significant increase of NSE (3.60 (2.66; 3.76) ng/ml in the “MET” group, 3.22 (2.94; 3.54) ng/ml in the “MET+EMPA» group, 2.72 (2.13; 2.72) ng/ml in the «Control» group) and NLC (4.50 (3.31; 5.56) ng/ml in the «MET» group, 5, 25 (3.75; 6.25) ng/ml in the «MET+EMPA» group comparing with 3.50 (2.25; 3.50) ng/ml in the «Control» group). Empagliflozin therapy led to a significant decrease in NLC already after 3 months (3.80 (3.25; 3.87) ng/ml), without significant influence on the NSE level. In the experiment, DM was characterized by an increased number of activated microgliocytes and destructured neurons and a decreased number of neurons with a normal structure. Empagliflozin therapy was accompanied by a decrease in the number of immunopositive microgliocytes in the CA1 zone of the hippocampus and an increase in the number of structured neurons.

**CONCLUSION:**

CONCLUSION: Type 2 diabetes mellitus is characterized by functional and biochemical changes in the central nervous system even under satisfactory glycemic control. Therapy with empagliflozin has a neuroprotective effect, manifested in an improvement in cognitive status and a decrease in NLC level. Empagliflozin reduces neuronal damage and abnormal microglial activation.

## ОБОСНОВАНИЕ

Сахарный диабет 2 типа (СД2) остается одной из ведущих проблем здравоохранения в мире. Среди причин смерти пациентов с СД2 сердечно-сосудистые события продолжают занимать лидирующие позиции, превалируя над чисто «диабетическими» причинами, такими как терминальная стадия диабетической нефропатии или диабетическая кома. Так, около 50% больных СД2 умирают именно от сердечно-сосудистых осложнений. Необходимо отметить, что в течение последних лет не инфаркт миокарда, привычно считавшийся наиболее грозным макрососудистым осложнением СД2, а нарушение мозгового кровообращения вместе с хронической сердечно-сосудистой недостаточностью (ССН) представляет собой наиболее частую причину смерти этих больных [1]. Наличие СД2 увеличивает риск развития инсульта более чем на 70%, а риск ишемического инсульта при СД больше в 2,27 раза по сравнению с таковым у лиц без СД [2].

Помимо острого нарушения мозгового кровообращения (ОНМК), наличие СД2 резко увеличивает риск развития хронической недостаточности мозгового кровообращения (ХНМК). Однако если частота ОНМК при СД известна и диагностика данного расстройства объективизирована, прежде всего широким внедрением методов своевременной нейровизуализации, ранняя диагностика и лечение ХНМК представляют трудность. ХНМК не вызывает развития очаговой неврологической симптоматики, однако негативно влияет на когнитивный статус, приводит к снижению качества жизни, инвалидизации и даже смерти больных. Одним из приоритетных направлений развития современной диабетологии может являться своевременное выявление данных нарушений с целью модификации терапии, в том числе сахароснижающей, с включением в нее препаратов, обладающих нейропротективным потенциалом.

Существующие отечественные [3] и зарубежные [4] алгоритмы ведения пациентов с СД2 ставят во главу угла не только и не столько способность антидиабетических препаратов снижать уровень гликемии, сколько сердечно-сосудистую безопасность («non-inferiority») или, что предпочтительнее, эффективность («superiority») препаратов. С этой точки зрения двумя приоритетными классами сахароснижающих лекарственных средств признаны ингибиторы натрий-глюкозного котранспортера 2 типа (иНГЛТ-2) и агонисты рецепторов глюкагоноподобного пептида 1 типа (арГПП-1). Так, представители этих двух классов способны снижать риск наступления общей конечной точки, уменьшать сердечно-сосудистую и общую смертность, а также риск развития инфаркта миокарда и госпитализации по поводу хронической сердечной недостаточности [5]. В то же время, по данным рандомизированных клинических исследований, среди широкого арсенала современных сахароснижающих препаратов только два обладают доказанной способностью уменьшать риск развития инсульта при СД — это длительно действующие парентеральные арГПП-1 семаглутид и дулаглутид [6]. Несмотря на то, что обобщенный анализ не показал способности иНГЛТ-2 снижать частоту инсульта в целом, появляются единичные данные об их потенциальном нейропротективном эффекте. Так, ряд представителей класса, прежде всего высокоселективные эмпаглифлозин (ЭМПА) и дапаглифлозин, способны положительно влиять на течение фибрилляции предсердий [7], тем самым создавая предпосылки к снижению риска кардиоэмболического подтипа инсульта [8]. В то же время сведения о влиянии современных антидиабетических препаратов, в том числе иНГЛТ-2, на течение ХНМК у пациентов с СД2 практически не представлены в мировой литературе.

Механизмы влияния иНГЛТ-2 на головной мозг также в настоящий момент являются предметом изучения. В эксперименте показано, что для высокоселективных иНГЛТ-2 характерно воздействие на систему mTOR, претерпевающую патологическую гиперактивацию при СД2 и других состояниях, сопровождающихся повышенным анаболизмом [9]. При ОНМК в эксперименте наиболее выраженный защитный эффект реализуют низкоселективные иНГЛТ-2 за счет дополнительной супрессии натрий-глюкозного котранспортера 1 типа, в том числе в головном мозге, что, в свою очередь, приводит к уменьшению ишемического-реперфузионного повреждения и ограничению очага некроза, а также снижению выраженности асептического воспаления [10]. Механизмы влияния иНГЛТ-2 на нервную ткань в условиях хронического нарушения мозгового кровообращения изучены неполно, в том числе, вероятно, в силу того, что влияние данного класса препаратов на ХНМК в целом продолжает изучаться, и однозначное подтверждение их нейропротективного действия в данных условиях пока не получено. До настоящего момента не определено, реализуют ли иНГЛТ-2 свое потенциальное нейротропное действие непосредственно на уровне нейронов или через влияние на состояние микроглии.

## ЦЕЛЬ ИССЛЕДОВАНИЯ

Изучить влияние терапии эмпаглифлозином на функциональные и лабораторные параметры повреждения ЦНС у больных СД2 и в условиях эксперимента исследовать механизмы нейротропного эффекта препарата.

## МАТЕРИАЛЫ И МЕТОДЫ

Исследование включало клиническую и экспериментальную часть. Клиническая часть ставила себе задачей феноменологически продемонстрировать наличие у высокоселективного иНГЛТ-2 ЭМПА нейропротективного эффекта при ХНМК и изучить взаимосвязь данного эффекта с уровнем контроля гликемии. Экспериментальная часть была направлена на прицельное изучение механизмов нейротропного эффекта ЭМПА при ХНМК на фоне СД2.

## Место и время проведения исследования

Место проведения. Клиническая часть исследования проводилась на базе НМИЦ им. В.А. Алмазова и ПСПбГМУ им. акад. И.П. Павлова. Экспериментальная часть исследования выполнялась в Центре доклинических и трансляционных исследований НМИЦ им. В.А. Алмазова и в Лаборатории функциональной морфологии центральной и периферической нервной системы Института экспериментальной медицины.

Время исследования. Набор пациентов в клиническую часть исследования и наблюдение за ними продолжались с декабря 2021 по июнь 2023 гг. Экспериментальная часть исследования выполнялась с марта 2022 по июнь 2023 гг.

## КЛИНИЧЕСКАЯ ЧАСТЬ ИССЛЕДОВАНИЯ

## Изучаемые популяции

В исследование были включены пациенты с СД2, получавшие монотерапию метформином (МЕТ). В случае, если пациенты имели целевой уровень гликированного гемоглобина (HbA1c) [3], что оценивалось исходя из их возраста и сопутствующей патологии, они были включены в группу «МЕТ» (n=19) — коррекция терапии не проводилась. В том случае, если HbA1c превышал целевой уровень не более чем на 2,5%, к терапии добавлялся иНГЛТ-2 эмпаглифлозин (эти пациенты составили группу «МЕТ+ЭМПА», n=20) на последующие 6 мес. Кроме того, была создана группа контроля, n=16, состоявшая из добровольцев в возрасте от 40 до 75 лет, не имевших СД, гипертонической болезни, ишемической болезни сердца, фибрилляции предсердий и других значимых сопутствующих патологий.

Критерии включения в группы «МЕТ» и «МЕТ+ЭМПА»:

Основные критерия невключения (для всех групп):

## Способ формирования выборки из изучаемой популяции

Выборка путем разбиения на группы.

## Дизайн исследования

Исследование представляло собой проспективное сравнительное нерандомизированное интервенционное исследование.

В группе «МЕТ» однократно, а в группе «МЕТ+ЭМПА» исходно, через 3 и 6 мес. выполнялись следующие исследования:

В группе контроля однократно осуществлялось общеклиническое обследование и определение концентрации маркеров нейронального повреждения, в связи с тем, что для данных маркеров отсутствует единый референсный интервал.

## Описание медицинского вмешательства

У больных с СД2 с HbA1c, превышавшим целевой не более, чем на 2,5% на фоне монотерапии МЕТ, производилось назначение эмпаглифлозина (Джардинс, Берингер Ингельхайм Фарма, Германия) в дозе 10 мг per os. Через 3 мес. осуществлялась повторная оценка уровня HbA1c — в случае, если целевой уровень был достигнут, то дозу эмпаглифлозина оставляли прежней. В случае недостижения целевого уровня HbA1с через 3 мес. дозу увеличивали до 25 мг.

При недостижении в группе «МЕТ+ЭМПА» целевого уровня HbA1c или снижении концентрации HbA1c менее, чем на 1,0% за 6 мес., сахароснижающая терапия корректировалась, в том числе с добавлением третьего препарата.

## Методы

Концентрации НСЕ и ЛЦН определялись в сыворотке крови иммуноферментным методом на анализаторе Cobas Roche с использованием наборов NSE Elycsis (Roche) и Bio-Techne соответственно.

## ЭКСПЕРИМЕНТАЛЬНАЯ ЧАСТЬ ИССЛЕДОВАНИЯ

Задачей экспериментальной части исследования стало изучение возможных механизмов нейротропных эффектов иНГЛТ-2 ЭМПА в условиях СД2.

## Изучаемая популяция и дизайн исследования

Исследование проводилось на крысах-самцах стока Wistar. В течение 4 недель и далее на протяжении эксперимента животные находились на диете с повышенным содержанием насыщенных жиров (22%). Через 4 недели от начала опыта внутрибрюшинно вводился раствор никотинамида, через 15 минут — раствор стрептозотоцина для моделирования СД [11].

Через 4 недели животные были разделены на группы: «СД» (n=5), в которой не назначалось никакое лечение, и «СД+ЭМПА» (n=4) — этим животным был назначен ЭМПА 2 мг/кг per os 1 раз в день на последующие 8 недель. Кроме того, была создана группа здорового контроля («Контроль»), n=5.

После введения стрептозотоцина, перед началом лечения и на протяжении 8 недель терапии, один раз в неделю осуществлялось определение гликемии.

Через 8 недель крысы были подвергнуты эвтаназии, и было произведено извлечение биоматериала головного мозга для последующего иммуногистохимического исследования.

## Описание медицинского вмешательства

Моделирование СД2 и верификация развития СД

Через 4 недели от начала эксперимента внутрибрюшинно вводился раствор никотинамида (Sigma-Aldrich, США) 230 мг/кг, через 15 минут — раствор стрептозотоцина (Sigma-Aldrich, США) 60 мг/кг. На 2-е и 3-и сутки после этого производилось определение гликемии с помощью глюкометра Accu Check Active путем пункции хвостовой вены. При выявлении в двух измерениях, выполненных в разные дни, гликемии, равной или более 11,1 ммоль/л, был диагностирован СД. При обнаружении меньших показателей гликемии хотя бы в одном из измерений выполнялся пероральный глюкозотолерантный тест: определение гликемии натощак и через 15, 30 и 60 мин. после зондового введения 40% раствора глюкозы 3 г/кг массы тела крысы. При выявлении в любой из точек гликемии, равной или больше 11,1 ммоль/л, диагностировался СД. В случае обнаружения меньших значений гликемии животные исключались из эксперимента.

## Методы

Проведение иммуногистохимического исследования

После эвтаназии во всех группах производилось извлечение головного мозга с сохранением мягкой мозговой и паутинной оболочек, фиксация в цинк-этанол-формальдегиде [12], обезвоживание и заливка в парафин. Морфологическому исследованию подвергали фронтальные срезы конечного мозга толщиной 5 мкм на уровне –3,36 мм±0,12 мм относительно брегмы. С целью оценки морфологического состояния нейронов зоны CA1 гиппокампа срезы окрашивали толуидиновым синим по методу Ниссля. Производилось выявление микроглиоцитов в структурах переднего мозга при помощи иммуноцитохимического маркирования с использованием козьих поликлональных антител к Iba-1 в разведении 1:1000 (Abсam, Великобритания). В качестве вторичных реагентов использовали набор VECTASTAIN Universal Quick HRP kit (Vector Labs, США). Пероксидазную метку выявляли с использованием диаминобензидинового хромогена (DAB+; Аgilent, США).

Статистический анализ

Статистическая обработка данных производилась при помощи программного пакета IBM SPSS Statistics-22 (IBM, США) и Statistica-10 (Statsoft, США). Статистический анализ производился при помощи непараметрических методов. Значимость различий между группами оценивалась с помощью непараметрического критерия Краскела-Уоллиса и Манна-Уитни для независимых выборок с применением непараметрического дисперсионного анализа (апостериорное попарное сравнение групп при помощи критерия Данна). Все показатели представлены в виде “медиана (25%; 75%)”. Значения p меньше 0,05 рассматривались как значимые.

Этическая экспертиза

Пациентам разъяснялась суть исследования, возможные риски и осуществлялось подписание информированного согласия. Исследование было одобрено этическим комитетом ПСПбГМУ им. акад. И.П. Павлова 22.11.2021 г. (протокол №11/2021) и этическим комитетом НМИЦ им. В.А. Алмазова 04.07.2022 г. (протокол №07-22-01С).

Все процедуры, выполненные с участием животных, соответствовали этическим стандартам, утвержденным правовыми актами РФ, принципам Базельской декларации и рекомендациям Комиссии по контролю содержания и использования лабораторных животных (IACUC) НМИЦ им. В.А. Алмазова. Исследование было одобрено IACUC, протокол ПЗ_22_2_СиманенковаАВ_V2 от 16.02.2022.

## РЕЗУЛЬТАТЫ

## Клиническая часть исследования

В таблице 1 представлены исходные характеристики пациентов групп контроля, «МЕТ» и «МЕТ+ЭМПА».

**Table:** Таблица 1. Исходные клинико-анамнестические характеристики пациентов Примечание: * — р<0,05, по сравнению с группой «Контроль». СД — сахарный диабет; ГБ — гипертоническая болезнь; ИБС — ишемическая болезнь сердца; ССЗ — сердечно-сосудистые заболевания.

Параметры	Контроль (n=16)	МЕТ (n=19)	МЕТ+ЭМПА (n=20)
Возраст, лет (Me (25%; 75%))	52,0 (47,0; 61,0)	64,0 (54,5; 67,0)	66,0 (58,5; 69,5)*
Анамнез СД, лет (Me (25%; 75%))	0	3,5 (2,0; 11,0)	5,5 (2,5; 9,75)
Наличие ГБ (n, %)	0 (0)	15 (78,9)	13 (65,0)
Наличие ИБС (n, %)	0 (0)	1 (5,3)	2 (10,0)
Наличие полинейропатии (n, %)	0 (0)	7 (36,8)	11 (55,0)
Наличие ретинопатии (n, %)	0 (0)	2 (10,5)	3 (15,0)
Наличие нефропатии (n, %)	0 (0)	0 (0)	1 (5,0)
Курение (n, %)	3 (18,75)	2 (10,5)	0 (0)
Отягощенная наследственность по СД (n, %)	4 (25,0)	6 (31,6)	6 (30,0)
Отягощенная наследственность по ССЗ (n, %)	10 (62,5)	7 (36,8)	4 (20,0)

Медиана возраста в группе «МЕТ» составила 64 года, при этом 11 человек (57,9 %) относились к пожилому возрасту, в соответствии с классификацией ВОЗ, в группе «МЕТ+ЭМПА» медиана возраста составила 66 лет, 12 человек (60,0 %) относились к пожилому возрасту, в связи с чем целевым для них следовало считать уровень HbA1c менее 7,5% [3].

Исходно в группе «МЕТ» уровень HbA1c был целевым у всех пациентов в соответствии с обозначенными выше критериями включения, а в группе «МЕТ+ЭМПА» уровень HbA1c превышал целевой (8,5 (7,7; 9,1) % (табл. 2).

**Table:** Таблица 2. Показатели гликированного гемоглобина и массы тела в группах «МЕТ» и «МЕТ+ЭМПА» Примечание: * — р<0,05, по сравнению с группой «Контроль», #— р<0,05, по сравнению с группой «МЕТ», & — р<0,05, по сравнению с предыдущим измерением.

	Контроль	МЕТ	МЕТ+ЭМПА
исходно	3 мес.	6 мес.
HbA1c, %	-	6,5 (6,1; 6,7)	8,5 (7,7; 9,1)#	7,2 (7,0; 8,4)#&	7,3 (6,7; 7,8)#
Масса тела, кг	73,5 (60,5; 85,0)	92,0 (78,0; 99,5)*	93,5 (83,5; 108,0)*	84,0 (83,5; 84,5)*#&	84,5 (83,0; 83,5)*#

Добавление к терапии ЭМПА уже через 3 мес. в целом привело к достижению целевых показателей гликемии, которые сохранялись и через 6 мес. При этом через 3 мес. приема ЭМПА в дозе 10 мг 14 пациентов имели нормализацию уровня HbA1c, а 6 пациентам потребовалось увеличение дозы ЭМПА до 25 мг. За 6 мес. терапии 4 пациента не продемонстрировали снижения концентрации HbA1c на 1% и более или достижения целевого уровня данного показателя, в связи с чем, по окончании исследования, их терапия была усилена введением третьего препарата.

Изначально пациенты с СД (групп «МЕТ» и «МЕТ+ЭМПА») имели большую массу тела, чем здоровые добровольцы группы «Контроль». Добавление к терапии ЭМПА привело к достоверному снижению массы тела по сравнению с исходным уровнем уже через 3 мес. и удержанию ее через 6 мес. (табл. 2).

Исходный уровень НСЕ и ЛЦН был достоверно выше у пациентов групп «МЕТ» и «МЕТ+ЭМПА», чем у здоровых добровольцев, причем различий между группами «МЕТ» и «МЕТ+ЭМПА» не было, несмотря на то, что пациенты группы «МЕТ» имели целевой уровень HbA1c. Комбинированная терапия МЕТ и ЭМПА не вызвала изменений в концентрации НСЕ, при этом привела к достоверному снижению концентрации ЛЦН уже через 3 мес. и поддержанию данного показателя на нормальном уровне, не отличном от такового в группе «Контроль» (рис. 1).

**Figure:**
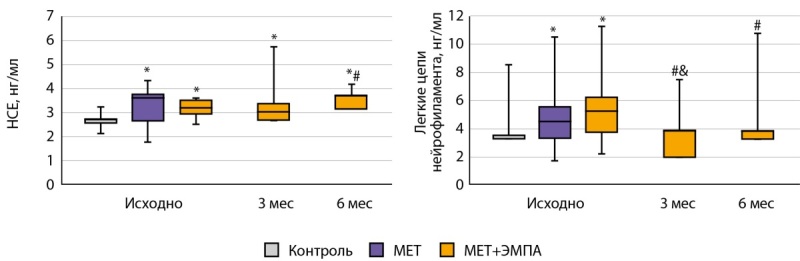
Рисунок 1. Уровень маркеров нейронального повреждения в группах «МЕТ» и «МЕТ+ЭМПА». * — р<0,05, по сравнению с группой «Контроль», # р<0,05, по сравнению с группой «МЕТ», & — р<0,05, по сравнению с предыдущим измерением.

Как у пациентов группы «МЕТ», так и у пациентов группы «МЕТ+ЭМПА», изначально наблюдалось снижение когнитивных функций при оценке с помощью шкал МОСА и MMSE (рис. 2). Добавление к терапии ЭМПА привело к улучшению когнитивного статуса уже через 3 мес., по данным шкалы MMSE, и нормализации показателей через 6 мес., по данным обеих шкал.

**Figure:**
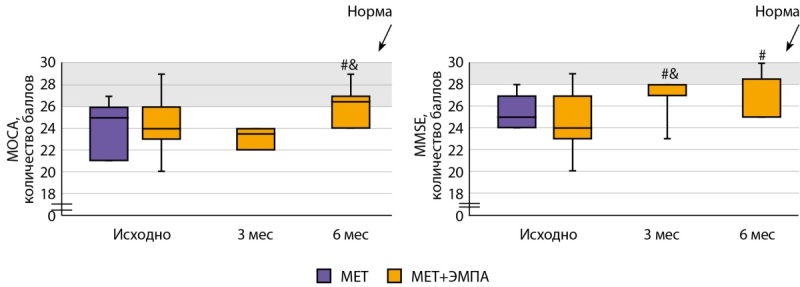
Рисунок 2. Показатели когнитивного статуса в группах «МЕТ» и «МЕТ+ЭМПА». # —р<0,05, по сравнению с группой «МЕТ», & — р<0,05, по сравнению с предыдущим измерением.

Корреляционный анализ не выявил взаимосвязи между выраженностью когнитивного дефицита и концентрацией маркеров нейронального повреждения, с одной стороны и длительностью анамнеза СД, уровнем HbA1c, массой тела — с другой. В то же время мы обнаружили отрицательную корреляционную связь между концентрацией ЛЦН и показателями когнитивного статуса, выраженными в баллах по шкалам МОСА и MMSE (r=-0,512, р=0,005 и r=-0,703, р=0,000 соответственно) (рис. 3).

**Figure:**
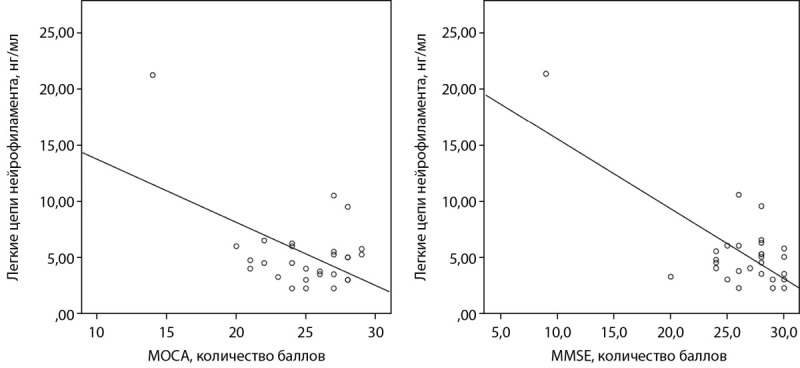
Рисунок 3. Корреляционная взаимосвязь между уровнем ЛЦН и показателями когнитивного статуса.

## Экспериментальная часть исследования

У 2 животных не удалось смоделировать СД, и количество крыс было увеличено с целью достижения исходно заявленного. Показатели гликемии животных на протяжении эксперимента представлены в таблице 3. Терапия ЭМПА позволила достичь удовлетворительного контроля гликемии.

**Table:** Таблица 3. Динамика гликемии (ммоль/л) крыс групп «Контроль», «СД» и «СД+ЭМПА» Примечание: СТР — стрептозотоцин, * — р<0,05, по сравнению с группой «Контроль», ¶ — р<0,05, по сравнению с группой «СД».

	После СТР (4 нед)	Перед лечением (8 нед)	9 нед	10 нед	11 нед	12 нед	13 нед	14 нед	15 нед	16 нед
Контроль	5,8 (5,3; 6,7)	5,5 (5,1; 7,1)	6,3 (5,4; 7,2)	5,7 (5,5; 6,1)	6,0 (5,5; 6,7)	5,7 (5,1; 6,0)	5,9 (5,1; 6,1)	5,9 (5,3; 6,1)	5,7 (5,1; 6,1)	6,4 (6,1; 6,9)
СД	17,6 (11,9; 21,2)*	13,9 (11,5; 16,1)*	13,1(12,2; 14,7)*	13,4(11,7; 17,2)*	13,0(12,5; 18,1)*	14,3(11,7; 14,7)*	13,2(11,9; 13,2)*	14,0(13,0; 14,7)*	12,8(11,9; 14,0)*	12,2(11,9; 12,9)*
СД+ ЭМПА	17,6 (11,9; 21,2)*	13,9 (11,5; 16,1)*	8,2 (7,8; 9,2)*¶	7,5 (6,8; 7,9)¶	7,17 (6,0; 7,2)¶	7,5 (5,9; 7,7)¶	7,1 (5,7; 7,6)¶	7,0 (5,9; 7,1)¶	7,2 (7,0; 7,7)¶	6,5 (6,2; 7,1)¶

При окраске на толуидиновый синий по методу Ниссля было выявлено, что количество нейронов с неизмененной морфологией в группе «СД» достоверно меньше, чем в группе «Контроль». Терапия ЭМПА привела к увеличению количества нейронов с нормальной структурой по сравнению с группой «СД», однако это число оставалось меньше такового в группе «Контроль» (рис. 4А).

Напротив, число нейронов, утративших нормальную структуру, в группе «СД» было достоверно больше, чем в группе «Контроль». Количество измененных нейронов в группе «СД+ЭМПА» было меньше, чем в группе «СД», и не отличалось от такового в группе «Контроль» (рис. 4Б).

**Figure:**
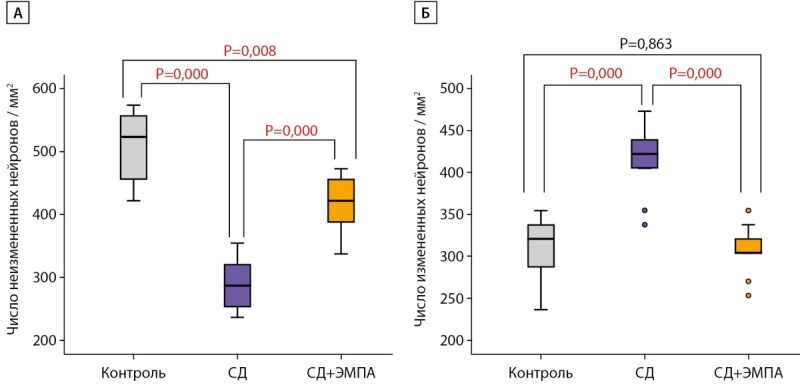
Рисунок 4. Число измененных и неизмененных нейронов в зоне СА1 гиппокампа крысы при окраске толуидиновым синим по Нисслю.

При исследовании препаратов, окрашенных по методу Ниссля, было установлено, что в группе «Контроль» наблюдаются нейроны с отчетливо выраженной, равномерно распределенной хроматофильной субстанцией, светлым ядром и метахроматически окрашенным ядрышком, располагающимся в центре ядра (рис. 5А). В группе «СД» в области CA1 гиппокампа регистрируются как гипо- так и гиперхроматозные нейроны. Практически на протяжении всей области CA1 наблюдаются нейроны, в цитоплазме которых имеются частичные признаки гиперхроматоза (рис. 5Г). В области перехода зоны CA1 в субикулюм выявляются нейроны с яркими признаками гиперхроматолиза. Наблюдается большое количество сморщенных темноокрашенных нейронов с неровными контурами. Здесь же встречаются клетки с просветленной цитоплазмой, отсутствием хроматофильного вещества, на этом этапе еще сохраняются ядро и ядрышко (рис. 5В). В единичных нейронах практически полностью отсутствует окрашивание на толуидиновый синий, как в цитоплазме нейронов, так и в ядре. Такой тип клеток принято называть клетками-тенями (рис. 5Б). Разрушение ядра служит достоверным признаком гибели нейрона. Также встречаются гипертрофированные нейроны, не имеющие видимых изменений. В группе «СД+ЭМПА» у большинства нейронов наблюдаются участки гиперхроматизации в цитоплазме (рис. 5Д, Е). При этом встречается и большое число нормальных нейронов. Клеток с яркими признаками гипер- и гипохроматоза не наблюдалось ни в одном из исследованных случаев.

**Figure:**
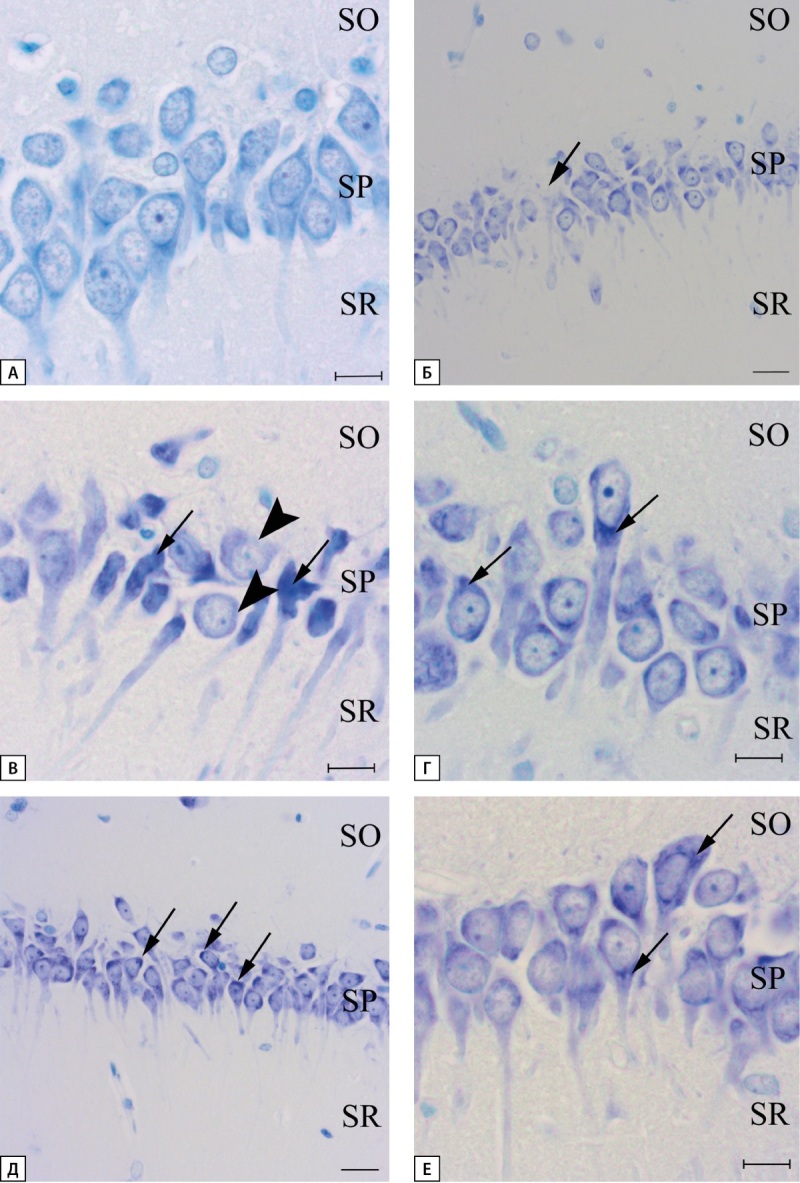
Рисунок 5. Морфология нейронов зоны CA1 гиппокампа крысы. Окраска толуидиновым синим по Нисслю. А — группа «Контроль», Б-Г — группа «СД», стрелкой отмечен нейрон с едва заметной окраской толуидиновым синим, так называемые клетки-тени (Б), рядом лежат клетки с признаками гиперхроматоза; гиперхроматозные, сморщенные нейроны с изъеденными контурами клетки, головкой стрелки отмечены нейроны с признаками гипохроматолиза (В); нейроны, с гиперхромными участками цитоплазмы (Г); Д, Е — группа «СД+ЭМПА», стрелкой отмечены гиперхромные области цитоплазмы отдельных нейронов. Масштабная линейка равна 10 мкм (А, В, Г, Е) и 20 мкм (Б, Д). SO-stratum oriens, SP-stratum pyramidale, SR-stratum radiatum.

Иммуногистохимическая реакция на Iba-1 выявила, что в группе «Контроль» клетки микроглии представлены рамифицированным типом и характеризуются небольшим округлым телом и длинными сильноветвящимися отростками. Тела клеток в плоскости среза встречаются редко и могут располагаться во всех слоях зоны CA1 гиппокампа (рис. 6А). В группе «СД» число микроглиоцитов значительно возрастает. Их тела увеличиваются в размерах, отростки становятся толще, что свидетельствует о переходной к активированной микроглии морфологии. В группе «СД+ЭМПА» число микроглиоцитов больше по сравнению с контрольной группой, но значительно меньше, чем в группе «СД». Здесь микроглиоциты характеризуются рамифицированной морфологией (рис. 6).

**Figure:**
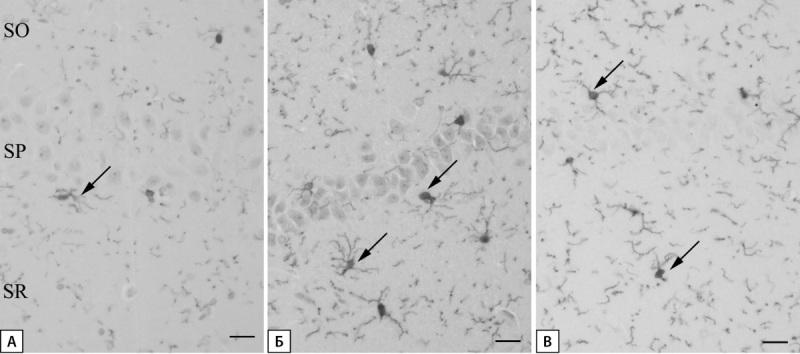
Рисунок 6. Микроглия области CA1 гиппокампа крысы. Иммуногистохимическая реакция на Iba-1. А — группа «Контроль», Б — группа «СД», В — группа «СД+ЭМПА». Стрелка указывает на тела микроглиоцитов. Масштабный отрезок равен 20 мкм. SO-stratum oriens, SP-stratum pyramidale, SR-stratum radiatum.

Наблюдалась статистическая тенденция (р=0,063) к большему числу иммунопозитивных клеток в группе «СД» по сравнению с группой «Контроль». Терапия ЭМПА привела к достоверному снижению числа иммунопозитивных клеток по сравнению с группой «СД» (рис. 7).

**Figure:**
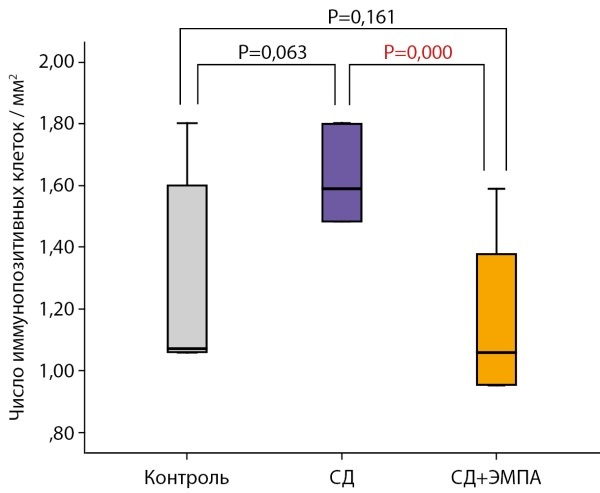
Рисунок 7. Число иммунопозитивных клеток в СА1 области гиппокампа, иммуногистохимическая реакция на Iba-1.

## ОБСУЖДЕНИЕ

## Репрезентативность выборок

Выборки, используемые в клинической части исследования, представляются репрезентативными, так как включают пациентов с СД2 без значимого ограничения по спектру сопутствующих патологий.

## Сопоставление с другими публикациями

Полученные нами результаты подтверждают, что СД2 сопровождается развитием патологических изменений в ЦНС, в том числе в условиях удовлетворительного контроля гликемии. Так, в нашем исследовании даже пациенты, имевшие целевой уровень HbA1c на монотерапии МЕТ, демонстрировали наличие когнитивной дисфункции, а также повышение уровня маркеров нейронального повреждения, НСЕ и ЛЦН.

Вариант повреждения ЦНС при СД по типу ХНМК описан в литературе. Одним из объяснений поражения ЦНС, несмотря на удовлетворительный контроль гликемии на момент обследования, может быть феномен «метаболической памяти» [13]. Так, известно, что ранняя интенсивная и одновременно безопасная сахароснижающая терапия позволяет предотвратить гликирование белков и липидов, образование активных форм кислорода, тем самым способствуя профилактике развития поздних микро- и макрососудистых осложнений. Напротив, длительно существующий неудовлетворительный контроль гликемии интенсифицирует указанные выше процессы, предрасполагая к развитию осложнений, в том числе со стороны ЦНС.

Другие механизмы формирования функциональных и биохимических изменений со стороны ЦНС при СД, вне прямой зависимости от контроля гликемии, могут сводиться к развитию асептического воспаления в ЦНС, нарушению целостности гематоэнцефалического барьера, а также нарушенному нейрогенезу. Известно, что процессы нейрогенеза в лимбической системе играют одну из ключевых ролей в обучении, запоминании информации. СД2 является независимым фактором риска замедления нейрогенеза и формирования несовершенных нейронных сетей [14].

Как упоминалось выше, в своем исследовании мы также показали, что СД2 характеризуется повышением уровня НСЕ и ЛЦН. Эти маркеры специфичны для поражения нервной ткани различного генеза: острого нарушения мозгового кровообращения, травматического повреждения, нейродегенеративного заболевания. Однако данные о повышении их при СД, в качестве маркеров ХНМК без инсульта, немногочисленны. Представлено несколько работ, описывающих взаимосвязь между уровнем НСЕ и выраженностью диабетической полинейропатии [15][16]. В 2023 г. были опубликованы результаты исследования, описывающие повышенный уровень НСЕ в сочетании с высокой концентраций С-реактивного белка в качестве предиктора развития диабетической периферической полинейропатии в течение ближайших 5 лет [17]. В литературе представлены также единичные сведения о повышении уровня ЛЦН у пациентов с СД2, опять же преимущественно в качестве индикатора диабетической нейропатии [18]. В то же время в 2023 г. S. Ciardullo впервые продемонстрировал, что СД2, безотносительно выраженности периферической нейропатии, ассоциируется с повышением ЛЦН, и что последнее коррелирует с тяжестью когнитивного дефицита [19].

Мы показали, что НСЕ и ЛЦН повышены у пациентов с СД2, при этом их концентрация не коррелирует с длительностью СД, степенью контроля гликемии и другими клинико-лабораторными параметрами, в то же время уровень ЛЦН отрицательно коррелирует с когнитивной функцией, определенной с помощью обеих использованных нами шкал, МОСА и MMSE. Более того, именно концентрация ЛЦН способна претерпевать динамику на фоне лечения, также одновременно с улучшением когнитивного статуса. В этой связи мы полагаем, что уровень ЛЦН может рассматриваться как маркер хронического повреждения ЦНС у пациентов с СД и служить в качестве метода ранней диагностики данного расстройства. Кроме того, этот показатель может обсуждаться в качестве маркера эффективности лечения, а именно реализации плейотропных нейропротективных свойств сахароснижающих препаратов.

Наше исследование продемонстрировало, что к таким препаратам, имеющим плейотропный защитный эффект в отношении ЦНС, может относиться высокоселективный иНГЛТ-2 ЭМПА. Так, мы показали, что добавление ЭМПА к терапии МЕТ приводит к улучшению когнитивной функции, что сопровождается снижением уровня ЛЦН. Полученные нами данные позволяют предполагать, что улучшение контроля гликемии на фоне комбинированной терапии МЕТ и ЭМПА вносит вклад в защиту ЦНС, однако может не являться ведущим. Так, достоверно более низкий уровень ЛЦН и лучший когнитивный статус в группе «МЕТ+ЭМПА» по сравнению с группой «МЕТ» при сходно удовлетворительном контроле гликемии в указанных группах позволяет предположить, что нейротропный эффект ЭМПА является самостоятельным и не обусловлен лишь положительным влиянием препарата на углеводный обмен. В пользу гипотезы о наличии у ЭМПА самостоятельного плейотропного эффекта в отношении ЦНС служит и отсутствие достоверной корреляционной связи между функциональными и биохимическими показателями ЦНС и клинико-анамнестическими характеристиками, такими как длительность анамнеза СД и уровень HbA1c. В то же время требуется дальнейшее динамическое наблюдение за пациентами с оценкой когнитивного статуса и маркеров нейронального повреждения, с тем чтобы определить, сохраняется ли выявленный нейропротективный эффект на фоне длительной экспозиции эугликемии. ЭМПА относится к высокоселективным иНГЛТ-2 и оказывает избирательное воздействие на натрий-глюкозные котранспортеры (НГЛТ) 2 типа, которые, как известно, преимущественно экспрессированы в почках. В то же время в последние годы появляется все больше сведений о наличии натрий-глюкозных переносчиков как первого, так и второго типов в ткани головного мозга и в эндотелии сосудов. В частности, НГЛТ 2 типа обнаружен в микрососудах гематоэнцефалического барьера, а также в области миндалевидного тела, гипоталамуса, ядра солитарного тракта [20]. Достаточно широкая представленность НГЛТ в различных регионах головного мозга создает предпосылки для реализации нейропротективного эффекта иНГЛТ-2. В то же время сами механизмы нейротропного эффекта данного класса препаратов остаются предметом изучения преимущественно в условиях эксперимента.

В ряде работ описан нейропротективный эффект ЭМПА на модели ишемического-реперфузионного повреждения на фоне экспериментального ишемического инсульта [21][22]. Данная модель представляется не вполне применимой в нашем случае, так как задачей настоящего исследования было изучение повреждения головного мозга в условиях ХНМК при СД. В эксперименте было показано, что ЭМПА предотвращает прогрессирование когнитивного дефицита у мышей с СД. Данное положительное влияние препарата, по мнению авторов, наиболее вероятно, связано с уменьшением выраженности оксидативного стресса и сопровождается усилением экспрессии мозгового нейротрофического фактора, BDNF [23].

Кроме того, одним из описанных механизмов влияния иНГЛТ-2 на ЦНС, и в особенности высокоселективного ЭМПА, может быть воздействие на систему mTOR. Известно, что состояния хронически повышенного анаболизма, к которым можно отнести ожирение и СД2, вызывают гиперактивацию данной системы [9], что, в свою очередь, приводит к развитию эндотелиальной дисфункции, усилению оксидативного стресса, прогрессированию воспаления, а также способствует нарушению целостности гематоэнцефалического барьера и даже вызывает в головном мозге нарушения инсулинового сигналинга, подобные таковым при нейродегенеративных процессах, в частности, при болезни Альцгеймера. Ингибирование НГЛТ 2 типа приводит к снижению патологической гиперактивации системы mTOR, тем самым нивелируя описанные выше процессы [9][10].

Результаты проведенного нами исследования подтвердили, что одним из механизмов нейропротективного действия высокоселективных иНГЛТ-2 является уменьшение выраженности асептического воспаления непосредственно в ткани головного мозга. Так, в эксперименте на животных мы показали, что СД2 сопровождается увеличением количества рамифицированной микроглии в СА-1 зоне гиппокампа по сравнению с группой здорового контроля. Применение ЭМПА позволяет уменьшить выраженность данных патологических изменений. С учетом того, что указанные изменения наблюдались на фоне достижения удовлетворительного контроля гликемии при помощи терапии ЭМПА, нельзя исключить положительное влияние улучшения гликемического профиля. В то же время ранее мы продемонстрировали, что подобный протективный эффект характерен именно для высокоселективных в отношении НГЛТ 2 типа препаратов [24] и практически не реализуется у низкоселективных иНГЛТ-2, несмотря на сопоставимо удовлетворительный контроль гликемии, что позволяет предполагать также наличие у ЭМПА самостоятельного положительного влияния на состояние микроглии. Кроме того, мы выяснили, что ЭМПА способен оказывать протективный эффект в отношении самих нейронов, что характеризуется обнаружением в данной группе нейронов с нормальной структурой, ядром, без признаков гипер- или гипохроматоза. Следовательно, мы можем предполагать, что защитный эффект ЭМПА реализуется как на уровне нейронов, так и на уровне микроглии и может быть лишь отчасти обусловлен улучшением гликемического профиля.

## Клиническая значимость результатов

Мы подтвердили, что СД2, даже при удовлетворительном контроле гликемии, сопровождается повреждением ЦНС, что требует активного и своевременного выявления, а также предложили лабораторный маркер, способствующий диагностике ХНМК при СД, который может быть использован в клинической практике. Мы показали, что ЭМПА обладает нейропротективным потенциалом, что позволяет рекомендовать его в качестве приоритетного препарата для лечения пациентов с СД и признаками ХНМК.

## Ограничения исследования

Ограничением исследования может быть включение в него только одного представителя класса иНГЛТ-2, что не позволяет делать выводы о том, является ли выявленное нейропротективное действие класс- или препарат-эффектом. Кроме того, необходимо дальнейшее динамическое наблюдение за пациентами для подтверждения того, что низкий уровень маркеров нейронального повреждения и удовлетворительный когнитивный статус сохраняются на фоне длительной экспозиции эугликемии. Наконец, дизайн экспериментальной части исследования не позволяет однозначно ответить на вопрос, каков вклад нормализации гликемического профиля в реализацию нейропротективного эффекта ЭМПА. В этой связи представляется целесообразным комплексное рассмотрение данного исследования, клиническая часть которого позволила подтвердить, что ЭМПА обладает самостоятельным плейотропным защитным эффектом в отношении ЦНС, а экспериментальная часть осветила подлежащие механизмы.

## Направления дальнейших исследований

Целесообразно проведение аналогичного по дизайну исследования с включением в него низкоселективных иНГЛТ-2. Кроме того, актуальным является сопоставление нейропротективного потенциала иНГЛТ-2 в условиях ХНМК с действием препаратов других классов.

## ЗАКЛЮЧЕНИЕ

Мы показали, что СД2, даже в условиях удовлетворительного гликемического контроля, приводит к развитию биохимических и функциональных нарушений со стороны ЦНС. Данные патологические изменения являются потенциально обратимыми и могут быть нивелированы назначением препаратов с нейропротективным потенциалом, какой, по результатам проведенного нами исследования, был доказан для высокоселективного иНГЛТ-2 ЭМПА. Механизм нейротропного эффекта ЭМПА, вероятно, обусловлен как непосредственным уменьшением повреждения нейронов, так и снижением патологической гиперактивации микроглии.

## ДОПОЛНИТЕЛЬНАЯ ИНФОРМАЦИЯ

Источники финансирования. Исследование выполнено за счет гранта Российского научного фонда №22-25-20163, https://rscf.ru/project/22-25-20163/ и гранта Санкт-Петербургского научного фонда, соглашение от 14.04.2022 №43/2022.

Конфликт интересов. Авторы декларируют отсутствие явных и потенциальных конфликтов интересов, связанных с содержанием настоящей статьи.

Участие авторов. Симаненкова А.В., Фукс О.С., Тимкина Н.В., Суфиева Д.А., Кирик О.В. — существенный вклад в получение, анализ и интерпретацию результатов, написание статьи; Коржевский Д.Э., Власов Т.Д., Каронова Т.Л. — существенный вклад в концепцию и дизайн исследования, внесение в рукопись существенной правки с целью повышения научной ценности статьи.

Все авторы одобрили финальную версию статьи перед публикацией, выразили согласие нести ответственность за все аспекты работы, подразумевающую надлежащее изучение и решение вопросов, связанных с точностью или добросовестностью любой части работы.
